# Promotion of physical activity in older adults: facilitators and barriers experienced by healthcare personnel in the context of reablement

**DOI:** 10.1186/s12913-022-08247-0

**Published:** 2022-07-27

**Authors:** Hanne Leirbekk Mjøsund, Lisbeth Uhrenfeldt, Elissa Burton, Cathrine Fredriksen Moe

**Affiliations:** 1 Faculty of Nursing and Health Sciences, Nord University, Universitetsallèen 11, 8026 Bodø, Norway; 2 Curtin School of Allied Health, Curtin University, Perth, Australia; 3 enAble Institute, Curtin University, Perth, Australia

**Keywords:** Health services of the aged, Interdisciplinary research, Exercise, Physical activity, Patient-centered care, Activities of daily living, Reablement, Sedentary behavior

## Abstract

**Background:**

Being physically active is important for maintaining function and independence in older age. However, there is insufficient knowledge about how to successfully promote physical activity (PA) among home-dwelling older adults with functional challenges in real-life healthcare settings. Reablement is an interdisciplinary, person-centered approach to restoring function and independence among older adults receiving home care services; it also may be an opportunity to promote PA. However, reablement occurs in many different contexts that influence how PA can be integrated within reablement. This study aimed to identify facilitators and barriers experienced by healthcare professionals (HCPs) that influence the promotion of PA within the context of reablement.

**Methods:**

This exploratory qualitative study is guided by a realist perspective and analyzed through inductive content analysis. Sixteen HCPs, including occupational therapists, physical therapists, registered nurses, and home care workers, participated in semi-structured interviews. The HCPs were recruited from four Norwegian municipalities with diverse sizes and different organizational models of reablement.

**Results:**

The HCPs experienced several facilitators and barriers at the participant, professional, organizational, and system levels that influenced how they promoted PA through reablement. Factors related to the individual person and their goals were considered key to how the HCPs promoted PA. However, there were substantial differences among reablement settings regarding the degree to which facilitators and barriers at other levels influenced how HCPs targeted individual factors. These facilitators and barriers influenced how the HCPs reached out to people who could benefit from being more physically active; targeted individual needs, desires and progression; and promoted continued PA habits after reablement.

**Conclusions:**

These findings exemplify the complexity of facilitators and barriers that influence the promotion of PA within the reablement context. These factors are important to identify and consider to develop and organize healthcare services that facilitate older adults to be active. We recommend that future practice and research in reablement acknowledge the variations between settings and consider mechanisms on a participant and professional level and within an integrated care perspective.

**Supplementary Information:**

The online version contains supplementary material available at 10.1186/s12913-022-08247-0.

## Background

The population is rapidly ageing [1], which has led to increased needs for assistance in daily living [2]. Global strategies call for innovative initiatives to ensure the sustainability of healthcare provision and promote healthy aging i.e., enhancing and maintaining the functional ability that enables well-being in older age [3].

Being physically active is important for maintaining functional ability and health in older age. Physical activity (PA) is commonly defined as “any bodily movement produced by skeletal muscles that requires energy expenditure” [4], and PA may be included within different types of activities, such as transportation, activities of daily living (ADLs), household activities, leisure activities, or specific exercises. The World Health Organization (WHO) recommends that older adults participate in moderate-intensity PA at least 150 minutes a week, in addition to completing activities targeting strength and balance and reducing sedentary time [4].

Despite strong evidence of the relationship between PA and function in older adults, PA levels are seen to decrease with age, particularly among people who depend on help from others to manage their ADLs [5]. Older adults who receive home care services report several barriers to being physically active, such as injury/illness, a feeling of being too old, and a fear of falling [6]. Although it is emphasized that healthcare professionals (HCPs) should provide evidence-based, simple, and timely advice about PA and sedentary behavior that is adapted to individual needs, capacity, and preferences [7], challenges remain about how this can be done in a meaningful and sustainable way in real-life healthcare contexts [8]. There is a need to develop approaches to promote PA that are effective both in the short and long term, meaningful for older adults, and reach people who need them [9]. More attention should be placed on developing interdisciplinary approaches and investigating how contextual factors influence PA promotion among individual older people, HCPs, and their practice and organizational systems [10].

*Reablement* is a person-centered concept of care that has been implemented in several countries over the last two decades. It may be a convenient arena for promoting PA among home-dwelling older adults experiencing functional problems. Reablement aims to improve function and independence for people receiving home care [11–13]. Participants recruited to reablement are typically older adults with a mean age of 80 years [14], though there is largely consensus that reablement should be an inclusive approach, irrespective of people’s age, capacity, diagnosis or setting [13]. By addressing goals prioritized by the individual, it builds on personalized plans involving the practice of daily activities, home modifications, use of assistive devices [13], and, to some degree, exercise components [14]. Reablement is typically delivered by an interdisciplinary team, with the involvement of different combinations of disciplinary groups, including occupational therapists (OTs), physical therapists (PTs), and registered nurses (RNs), in addition to home care assistants or other staff from the home care service [14]. OTs, PTs, and RNs typically have the primary responsibility for conducting assessments and developing and adjusting the reablement plan, while the responsibility for delivering reablement on a day-to-day basis is delegated to staff from the home care services [15, 16]. However, the context of reablement differs, often involving different disciplinary groups, task allocations, and collaborative approaches [14, 17, 18]. In the following, HCPs will be used as a common term for all healthcare professionals working with reablement, while the term home care staff will be used for the staff from the home care organizations working with the participant, which may include home care assistants, RNs, or other professionals. The term ‘participant’ will be used for older people who receive reablement.

Although PA is an essential factor for improving and maintaining function in older age, there is little evidence of how reablement influences older adults’ PA levels [14]. A recent Delphi study among international reablement experts found diverse perspectives on whether or not exercise or motivation to increase PA should be included in reablement, and fewer than half of the experts agreed that exercise and motivation to increase PA should be part of the reablement concept [13]. Similarly, a recent study by our research team, that built upon the same interviews as the current study, found that HCPs working in reablement in a Norwegian context had diverse perspectives on *how* PA should be integrated within reablement [19]. The HCPs had a shared overall perspective that PA involved all types of physical activities, and that daily activities were a core type of PA in reablement. However, while some HCPs considered PA a central part of reablement to improve the participants’ physical function, other HCPs did not focus on PA particularly; they rather saw it as a positive consequence of participating in meaningful activities in daily living [19]. To embrace the HCPs’ differing perspectives on PA, we will in the following consider promotion of PA to include general facilitation of activity in daily living, including both everyday activities and PA/exercises particularly targeted physical capacity. Although the HCPs’ differing perspectives on PA may complement each other in the delivery of interdisciplinary and person-centered reablement, several studies have found that the approaches and activities prioritized in reablement differ between settings [19–21]. It has been suggested that contextual differences between or within countries may explain the different perspectives and priorities in reablement [12–14, 19, 21].

The context of reablement can relate to different aspects of professional practice and may involve factors on micro (i.e., factors related to individual participants), meso (i.e., factors related to HCPs professional practice and organization of that practice), and system (i.e., factors related to healthcare system/policies) levels [22]. These levels may include different facilitators and barriers influencing how reablement is delivered, from specific factors influencing an individual in a particular situation to more generic factors influencing several aspects of reablement delivery. To deliver person-centered care, services need to be delivered in an integrated way, requiring continuity and collaboration between the different levels and sites within the healthcare system [22–24]. In the context of reablement, no studies have identified the factors that influence how HCPs can support participants to become more physically active in daily living. Therefore, this study aimed to identify facilitators and barriers experienced by HCPs that influence the promotion of PA in the context of reablement.

## Methods

This study is a qualitative exploratory study based on individual interviews, from which one study has been published previously describing some of its methods [19]. The study design is inspired by a realist perspective, focusing on gaining an increased understanding of mechanisms that may explain why reality unfolds as it does in a particular context [25]. To ensure that the relevant study information is reported, we followed the consolidated criteria for reporting qualitative research (COREQ) [26].

### Study context

In Norway, where this study was conducted, reablement is delivered free to participants through publicly funded healthcare services. Municipalities are obligated to deliver care that meets national laws and overall policy. However, they have the authority to organize and deliver the services in whatever way they choose. Reablement has been rapidly and extensively implemented in Norway over the last decade, though with significant differences in its organization and delivery [21, 27]. Two main organizational models have been identified, in which reablement is either provided as an integrated part of home care services or through a specialized reablement team [27]. The implementation of reablement has been supported by national healthcare policies [27], and it is suggested as one of several strategies within a national quality reform currently being implemented in Norwegian municipalities to provide services that help older adults maintain their independence in daily life and encourage a safe and active older age [28].

### Sampling strategy and recruitment

A purposive sampling strategy was used based on principles of variation sampling, in which the intention is to reach variation in small samples based on pre-defined selection criteria [29]. To gain variation at the municipal level, we selected four municipalities that provided reablement, and that differed in size and organizational model because this may involve different premises for practice [21, 27]. To gain variation at the HCPs’ level, we included HCPs (*n* = 16) with diverse professional backgrounds who were central in delivering reablement in their respective municipality. The HCPs had to have at least 1 year of experience with reablement. By including this heterogeneity in the study sample, we aimed to gain knowledge of central factors that cut across the existing variation and also captured diverging factors influencing how HCPs promoted PA within their context.

Eligible municipalities were selected, and the leaders of the reablement teams in these municipalities were initially asked for permission to contact potential candidates on their team. The leaders were encouraged to suggest potential candidates who were reflective of their practice, and represented diverse professional groups. Each potential candidate was contacted in person by phone or e-mail, given verbal and written participant information, and signed a consent form before any data collection. All the reablement leaders contacted were positive about participation, and all the HCPs who were recommended and contacted agreed to participate.

### Data collection

The research team developed a semi-structured interview guide and discussed it with HCPs working with reablement in a municipality not included in the study (see online additional file 1). The interview guide served as a guide for conversational topics and direction throughout the interviews, but the question order was not followed strictly.

Each HCP participated in one interview. Each interview lasted 70–90 minutes and was conducted by the first author (HLM), who had no prior relationship with the HCPs. Before the interviews, the interviewer gave brief information about her professional background and the aim of the study. The interviews were undertaken as individual face-to-face interviews between May and October 2019 in a quiet office or meeting room at the participants’ workplace and were audio-recorded.

### Data analysis

**Fig. 1:**
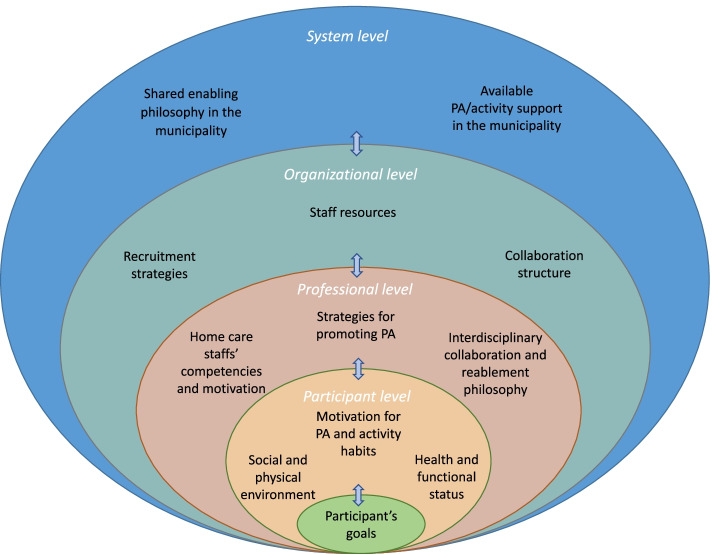
Factors experienced by HCPs to influence PA promotion through reablement. The Figure illustrates factors experienced by HCPs that could fall out as either facilitators or barriers for promoting PA through reablement. This involved an interdependent coherence between factors on different levels, including a participant, professional, organizational-, and system level. Abbreviations: HCP = Healthcare professional, PA = Physical activity

We used an inductive qualitative content analysis, informed by Erlingsson and Brysiewicz [30]. Interviews were transcribed verbatim and read several times, noting reflections and main impressions accordingly. Each transcript was then systematically searched for units of text about facilitators and barriers that influence how HCPs promote PA and given codes using NVivo software©. The text units were condensed and organized into categories. This initial stage of the analysis demonstrated great variability and complexity of different factors influencing how the HCPs promoted PA in reablement. To better structure the continued analysis, we divided the categories we had identified into a participant,- professional,- organizational,- and system level, inspired by the integrated care mechanisms framework by Valentijn et al. [22]. Followingly, we continued organizing and questioning the content and coherence between the categories, as well as clarifying facilitators and barriers within each category. An overview of the categories, organized within each level is illustrated in Fig. 1. This was an interpretative, non-linear process involving careful consideration of the consistency between parts of the data and the interpretations achieved through the analysis. The analysis was undertaken by one researcher (HLM) and was critically discussed among the research team to analyze the coherence of the findings and how the researchers’ preunderstandings influenced the analysis. After analyzing 16 interviews, we found the data to be sufficiently saturated for this study. We found that the HCPs reported factors within the same overall topics yet described variations in how these factors influenced their practice. This approach followed the principles of data saturation within a reflexive content/thematic analysis approach [31]. We used the questions raised in the checklist developed by Elo et al. [32] to critically reflect upon the trustworthiness of the study’s methodology. Quotes from the interviews are presented to exemplify the main findings. The quotes have been translated to English and edited slightly to improve grammar and flow, but their meaning and intent have not been altered.

## Results

### Study participants

Sixteen HCPs participated in this study, including four OTs, four PTs, four home care assistants, two RNs, and two HCPs with other health and/or social educational backgrounds (their particular education is not specified to avoid compromising their confidentiality). The HCPs’ median age was 46.5 (range 29–57), and two of them were male. On average, they had 19 years of professional experience (range 4–33) and 4 years of experience working with reablement (range 1–6 years). An overview of the characteristics of the participants is presented in Table 1.

**Table 1:** Characteristics of healthcare personnel

Healthcare personnel	Number	Gender (male/female)	Age, Mean (range)	Years of professional experience, Mean (range)	Years of experience with reablement, Mean (range)
OTs	4	1/3	36 (29–43)	11.5 (6–17)	4.5 (4–5)
PTs	4	1/3	51 (40–56)	24 (17–31)	4 (1–6)
RNs	2	0/2	51 (44–57)	17.5 (4–31)	3.5 (3–4)
Home care assistants	4	0/4	54 (49–56)	30.7 (29–33)	4.5 (4–6)
Other	2	0/2	33 (30–35)	9.5 (7–12)	3.5 (3–4)

*Abbreviations*: *OT* occupational therapist, *PT* physical therapist, *RN* registered nurse

### Reablement settings

The HCPs were employed in four different Norwegian municipalities varying in population (4000–200,000). Two of the municipalities had organized reablement as an integrated part of the home care services, in which OTs and PTs from the rehabilitation section collaborated with staff from the home care services. According to their shift schedules, the home care staff could either be a few selected HCPs from the home care services trained in reablement or any staff from the home care service. In the other two municipalities, reablement was delivered by specialized reablement teams involving HCPs employed on the team. One of these specialized teams consisted of a PT, an OT, and two home care assistants. The other specialized team consisted of only PTs and OTs and involved HCPs from the home care services when deemed appropriate. The duration of the reablement interventions in all municipalities was approximately 6 weeks, but if needed, these could increase. The frequency and duration of visits were variable between municipalities, ranging between 2 and 5 visits per week and 20–60 minutes per visit. General characteristics of reablement in each municipality are presented in Table 2.

**Table 2:** General characteristics of reablement organization in each municipality

	Municipality 1	Municipality 2	Municipality 3	Municipality 4
Organizational model	Integrated	Integrated	Specialized team	Specialized team/integrated
Duration of reablement intervention	~ 6 weeks	~ 6 weeks	~ 6–8 weeks	< 6 weeks
Visits per week	2	2–5	2–3	5
Duration per visit	~ 30–60 min	~ 20 min	~ 60 min	~ 60 min
HCPs involved	PT, OT, and selected home care staff with reablement training/experience (RNs and home care assistants)	OTs, PTs, and general home care staff (RNs, home care assistants, others)	PT, OT, and home care assistants	PTs, OTs, and home care staff involved occasionally
Eligibility for receiving reablement	Discretionary judgments by HCPs - Motivation - Goal of improving daily activities - No need for specialized rehabilitation	Standardized criteria - ADL and cognitive score within set limits - Motivated for reablement - Excluding people in the palliative phase or with extended drug or psychiatric problems	Discretionary judgments by HCPs	Standardized criteria - Motivation and ability to participate in reablement five times a week - Being able to set goals for themselves
Referral procedure to reablement	Participants apply themselves	Only home care staff can refer people to reablement	Anyone can refer/apply	Anyone can refer/apply

*Abbreviations*: *HCP* Health care professional, *OT* occupational therapist, *PT* physical therapist, *RN* registered nurse, *HT* home trainer, *ADL* activities of daily living

### Facilitators and barriers for promoting PA

The HCPs experienced several factors at a participant, professional, organizational, and system level that influenced how they promoted PA in the reablement context, as illustrated in Fig. 1. The degree to which these factors were experienced as facilitators or barriers differed between reablement settings and depended on the interrelationship between factors on different levels. An interdependent coherence between facilitators and barriers on all levels influenced how the HCPs recruited people who could benefit from being more physically active; targeted PA to the individual participants’ desires, needs, and progress; and facilitated continued long-term PA habits. Some facilitators and barriers experienced by the HCPs influenced their ability to promote PA as well as their reablement delivery in general.

#### Facilitators and barriers at a participant level

The participant level included factors related to the individual participant that influenced how the HCPs promoted PA, including the participants’ goals, motivation for PA and activity habits, health and functional status, and physical and social environment.

##### Participant level: participants’ goals

The HCPs emphasized that promoting PA should be closely related to the participants’ goals. Having clear and meaningful goals was considered facilitative for introducing PA in a meaningful way. As one HCP noted, “*The participant needs to be determined that this is something they want. [ …*] *This is something they want to achieve” (PT, 11).* While some participants had clear goals, the HCPs also encountered participants who found it difficult to set specific activity-related goals: “*Many are like...yes, I just want to become … stronger in the legs, right? [ …] But what do you want to USE that for then?” (PT, 15).*

##### Participant level: motivation for PA and activity habits

The participants’ motivation for PA was considered key to how the HCPs promoted PA. As one HCP noted, “*What it takes to succeed [to promote activity]? They need to be motivated, simply. And then they need to be motivated to do some self-efforts [...] in order to be able to continue after we have finished the period” (home care assistant, 3).* Having previous positive PA experiences and PA habits in daily living was considered a facilitator, along with the participants understanding how PA habits influenced their function. As noted by an HCP, “*If a participant has been taking walks every day or every second day and has been going to some kind of exercises and [ …] has a SOCIAL activity away from home, we often succeed VERY well with those kinds of participants” (OT, 14).*

The HCPs further believed they had more success in re-establishing PA through meaningful activities that the participants had recently engaged in, rather than activities they had not partaken in for a long time: “*Often, it is a bit about how long they have been passive. The longer they have been passive, the more difficult it may be to get them going again” (Other, 4).*

##### Participant level: health and functional status

The HCPs suggested that the participants’ health and functional status, such as medical conditions, hospitalization, falls, pain and cognitive function, could be barriers to promoting PA. One PT observed, “*It is essential for their progress that the participants remain healthy, that they do not experience new falls, and that they start eating and drinking what they need to engage in reablement in a good way” (PT 1.)* They also noted that anxiety and fear of falling were common barriers to being active: “*We have more and more participants that are anxious. [ …] They are afraid of going outside and afraid of falling. They often remain at home, and then they become inactive and passive, which again make them weak and fragile” (OT, 14).*

##### Participant level: social and physical environment

The participants’ social environment could facilitate and impede promoting PA, and existing beliefs from people in their social environment regarding function and activity in older age were considered an essential factor. People in the participants’ social networks could be important supports for motivating and enabling the participants to be physically active. “*His wife was involved and supportive. [ …] He had begun to walk the stairs a lot and took the stairs rather than the elevator when he visited his daughter. And they [wife and daughter] were involved and motivated him to do these things” (OT, 8).* However, family members could also restrict the participants from being active by constraining them from participating in activities they considered harmful or by doing the activities for them, rather than letting them do things themselves. One HCP noted, “*What we often see, unfortunately, is that the family members want to help their parents, so they take some of their tasks.” (PT, 15)*.

The participants’ physical environment could also be a facilitator or barrier to promoting PA. Some challenges within their current physical environment were considered important for maintaining meaningful PA. The HCPs were therefore skeptical about a trend of rearranging for easy living in older age: “*They may move to a block apartment because they believe that when they become old, they will stop walking stairs because it becomes too exhausting [ …] And then they become sedentary in that apartment.” (PT 15).* However, challenges in the physical environment, such as steep stairs, long distances or climate, could also be barriers to being physically active and prevent the participants from participating in the activities they preferred: “*There are many [participants] that cannot get out. [ …] When it is about walking outside or … walking to the trashcan, mailbox and those things, then it can easily become unsafe with ice and slippery [ground]” (OT, 2).*

#### Facilitators and barriers at a professional level

The professional level included factors related to the HCPs’ practice, such as the HCPs’ PA promotion strategies, their reablement philosophy and interdisciplinary collaboration, and the home trainers’ competencies and motivation.

##### Professional level: strategies for promoting PA

The HCPs believed that their strategies for promoting PA were essential in supporting the participants in developing new PA habits. The HCPs emphasized different strategies for promoting PA through reablement, including physical exercises or PA through daily activities and more or less standardized approaches. Some HCPs described how they often preferred standardized exercises that they knew improved function: “*The ‘Hellbostad exercises’ are often used because they are well documented” (PT, 5).* Some of the HCPs pointed out that the exercises had to be simple and easy to understand for those who were to follow up, and they, therefore, preferred standardized exercises “*because it should [ …] [involve] easy exercises that do not require particular competencies” (Other, 4).* However, the HCPs were not always confident that such exercises were sufficiently targeted to the participants’ individual needs. A nurse noted, “*Sometimes it has occurred to me that this is a person that is as light as a feather and jumps off the chair … and here they do 20 knee bends and get up and down from their chair. Perhaps we should have included some weights [ …] or heavier exercises” (RN, 6).*

The HCPs noted that many participants could be motivated to do exercises when HCPs supervised them, but they were doubtful that such exercises were continued after reablement ceased. An OT stated, “*And that is what they succeed with the most when they are to continue over time, that they have something that is important and meaningful for them. [ …] There are not that many of them that bother doing exercises day in and day out.” (OT, 14).* To promote continued PA habits for the participants, they had the most success with encouraging them to add PA through daily and familiar activities, such as walks, stair walking, housework, and other meaningful physical activities that the participants were motivated to do: “*It is about motivating them to do something between the [reablement] visits. And [I] continuously talk about how important it is [...] to try walking the stairs, vacuuming, those things they should have done” (PT, 1).*

##### Professional level: interdisciplinary collaboration and reablement philosophy

The HCPs suggested that their interdisciplinary collaboration was essential to improve facilitators and remove barriers for PA among the participants, according to their individual needs and desires. The HCPs strongly emphasized the advantages of having HCPs with different competencies involved in reablement to see things from different perspectives and involve those with the necessary expertise: “*That’s what’s so good when you do such an assessment with different disciplinary groups all together because we are wearing different glasses when we go in. But when we sit together, I feel that we are quite in tune about the goals that we have with the participant.” (PT, 1).* Although embracing different professional approaches, some HCPs acknowledged that they lacked a shared reablement philosophy in their team, which was a barrier to working collaboratively toward the participants’ goals. The HCPs indicated how there were different perspectives with regards to how PA should be integrated in reablement; whether or not it should only be included if it was part of the participants’ goal activities; include particular exercises or how PA should be progressed. An OT observed, “*We have very different backgrounds. [ …] It is not that we haven’t tried creating a common basis, but there is something about UNDERSTANDING that basis, that everyone understands it in the same way. [ …] We need to be in unison on the BASIS, and that’s what’s so complicated with teamwork.” (OT, 12).*

A close and respectful interdisciplinary collaboration between the HCPs was considered important to learning from each other, developing a shared reablement philosophy, and sharing tasks adequately between them. Also, the HCPs found it important to communicate the progression or adaptation of activities between them to adequately meet the needs of the participants: “*It is important to have good documentation of the exercises so we can see if there is any progression or increased pain or something like that in order to follow up* “*(Other, 7).*

##### Professional level: home care staffs’ competencies and motivation

The home care staff’s competencies involved in reablement were viewed as essential for promoting PA. It was considered a facilitator if the home care staff knew the particular participant, had additional training in reablement or rehabilitation, and had significant experience with reablement: “*It is a huge advantage to have the home care services so close with us because they have known them [the participants] over a long time, perhaps before their balance started to weaken. They know what they could do before and what they liked doing before. That’s what’s so very good with our home trainers [home care staff involved in reablement]—that they are the same that have been involved all the time. Then they have become good at this” (PT, 1).*

However, some HCPs suggested that the home care staff who delivered reablement did not always have the necessary competencies or motivation, which could be a barrier to promoting PA: “*Many assistants have three days of training [ …*]. *They do not have this background to see the entirety: that it is very important that this person gets to do things themselves” (home care assistant, 16).* Also, “*there are many here [in the home care service] that find reablement boring” (RN, 6).* Having previous successful experiences with promoting PA was believed to facilitate home care staff to become motivated to continue promoting PA.

#### Facilitators and barriers at an organizational level

The organizational level involved factors related to how reablement was organized, including recruitment strategies, staff resources, and collaboration structures, which had influence on how the HCPs were able to promote PA.

##### Organizational level: recruitment strategies

The recruitment strategies for reablement were essential for how the HCPs believed they could reach out to people who could benefit from being more physically active. The HCPs emphasized the importance of reaching out to people with early signs of functional decline or recently reduced activity levels. One PT stated, “*We should be able to get in touch with those who just start deteriorating a bit functionally—those who have stopped walking outside, stopped walking to the grocery shop, started receiving domestic help.” (PT, 15).*

Having reablement organized as an integrated part of the home care services was believed to improve the ability to recruit eligible participants by improving the home care staff’s knowledge about reablement and their awareness and ability to identify people early who had started to become more passive in daily living activities: “*There are quite a few from the home care staff that have become experts in observing and identifying potential participants” (PT, 5).* However, when reablement was organized as a specialized team, the HCPs found it challenging to reach out to the people they believed could benefit the most from reablement: “*I don’t feel that we reach out to that many. A few people in the municipality receive a really good service when we visit them, but I believe that there are more people out there that could have needed [reablement]” (home care assistant, 13).*

The HCPs emphasized that the availability of reablement needed to be known in other healthcare services and society, in general, to reach out to eligible participants who may benefit from it. Also, having a clear conceptualization of reablement and well-defined eligibility criteria was considered important to ensure that suitable candidates were recruited to reablement. Some of the HCPs emphasized that it was important to clarify that reablement was not only an exercise program, but involved a broader activity approach. One HCP noted, “*We need to be able to better communicate who we are and what reablement is to the leadership in the municipality, collaborative HCPs, and the community population [ …] so we can be used in a more constructive way” (PT, 11).*

##### Organizational level: staff resources

The available staff resources were closely related to how reablement was organized and was considered important to how the HCPs could meet the participants’ needs and support them to become more active. The HCPs emphasized that staff stability was important in developing the competencies required to promote activity. However, when reablement was organized as an integrated part of the home care services, some HCPs experienced a high turnover of home care staff and suggested it could be a barrier for developing the home care staffs’ competencies: “*There is a high turnover of staff in the home care service. And then it is also a challenge to, among other things, give all of the staff good training in what reablement is because not everyone knows” (Other, 7.)*

Also, the time available for reablement differed between the municipalities, influencing how PA could be promoted. Some of the HCPs found time restrictions within the home care services limited their abilities to do the activities they believed were important for the participants: “*The time can be a barrier [if] the home care service can allocate 15, maximum 20 minutes, right, in every visit. [ …] If the goal is to become more confident when walking outdoors, and this is in the winter season and … from the [time] from the home care service meet up until the person [the participant] has put on clothes and shoes, then it has been 8 minutes, right” [PT, 5].*

##### Organizational level: collaboration structure

To motivate the participants to be active and progress their activities appropriately, it was considered important to have a collaborative structure that enabled regular interdisciplinary meeting points in which the HCPs could learn from each other and discuss how to approach each participant. However, some HCPs experienced insufficient opportunities for such meetings, which was a barrier to collaboration: “*There is no time for us to meet, only us home trainers and perhaps with OT and PT” [home trainer, 3].* Some HCPs emphasized how informal conversations and being located in the same building facilitated interdisciplinary collaboration. A PT observed, “*It is very favorable for us that we are located in the same building. [ …] We meet each other almost every day, and then it is easy to think that … perhaps we should have had reablement for her” (PT, 1).* When reablement was organized as an integrated part of the home care services, some HCPs found it challenging to establish times to meet that were suitable for all: “*The logistics are difficult, really difficult. [ …] First of all, we are limited to using the time after lunch for meetings with the participants and the home care service. [ …] The aim is to have all three professional groups [OT, PT, primary contact from the home care service] involved all the time, but it is difficult” (OT, 8).*

While the HCPs emphasized the importance of getting to know the participants to promote PA in a meaningful way, the organization of reablement influenced how the HCPs were able to continuously follow up the participant during reablement. Some HCPs found it useful to involve a few different home care staff because they had different approaches to how to motivate the participants: “*It is beneficial that we have several [home] trainers because we see things differently, right? And we communicate a bit differently. Then you are a bit more tuned in each time. If you are the same, you can become a bit tired of repeating yourself” (home care assistant, 3).* However, some HCPs experienced a low continuation of staff. It often involved different home care staff delivering reablement from day to day, which made it difficult to build a relationship with the participant and support them in progressing their activities in a meaningful way. A nurse noted, “*I think it could have been beneficial to have a defined group visiting each participant. Not a person that never has been to the participant before and [says], ‘Yes, let us do some exercises’” (RN, 6).* When different home care staff were involved, the HCPs found it essential to communicate what was done at each visit, to ensure appropriate progression of activities. However, this was often challenging: “*It demands quite a lot from us and the collaboration with the home care service [ …] And if the one [home care staff] coming in does not know what was done yesterday, it becomes difficult to progress that” (OT, 8).*

#### Facilitators and barriers at a system level

The HCPs also experienced factors on a municipal system level that influenced promoting PA with participants through reablement. The degree to which the municipality was working from a shared enabling philosophy was considered essential, along with having available and varied activity support in the community.

##### System level: shared enabling philosophy in the municipality

The HCPs suggested that having a shared enabling philosophy implemented into the municipal health and home care services facilitated their ability to adequately support participants to be active: “*Enablement is the overarching umbrella for everything that goes on in this municipality. [ …] [Enablement is] … the philosophy … that whatever you are able to do in an activity, you should be allowed to do” (PT, 5).* Integrating an enabling philosophy was considered important for identifying and recruiting people in the community who could benefit from becoming more active, facilitating the collaboration between reablement and other healthcare services, and providing the necessary activity support after reablement. An HCP stated,” *It is important that we [the home care service] follow up on what they have trained [in] and that we do not return to helping [doing for] so much” (home care assistant, 16).*

Most HCPs experienced that an enabling philosophy was not sufficiently implemented in their municipalities, which they believed was a barrier to promoting PA: “*We do actually have a role out in society regarding implementing enablement [an enablement philosophy], right? But … we are not there yet. [ …*]” *(home care assistant, 10).* The HCPs believed that the existing organization—available resources, leadership, and mindset within the healthcare system—was a barrier to successfully implementing this philosophy: “*Reablement was supposed to be a little [method] … to drift the home care services in another way. That rather than receiving services, they should receive exercise. [ …] We [reablement] were supposed to change the entire home care services, change their attitudes. [ …] But then they need to … First of all, they need to have the time for that. And secondly, they need to understand that this is for the best for the participant” (PT, 15).*

##### System level: available PA/activity support in the municipality

Having available PA support and other activity offers in the municipality was considered critical to facilitating continued PA among the participants: “*They [some of the participants] need follow up over a longer period of time. We are short and intensive, right, so they do get a boost. But then they need to have someone to continue following them up” (OT, 14).* It was considered a facilitator for promoting PA if the municipality had varied and easily accessible activity offers that could meet different needs and desires among the participants. Also, the HCPs found it important to introduce such activities to the participants during or immediately after reablement to support the participants’ confidence to engage in the activities: “*Sometimes we have chosen to do some of the exercises we do here [in the exercise groups] at home with them. So they know what kind of exercises they will do when they come here. [ …] We aim to make them confident and show that they are capable enough, strong enough, and fit enough and such. [ …] So it is actually the same person [PT] that continues the exercises” (PT, 1*).

However, some HCPs experienced a lack of available activity opportunities that targeted different needs and desires of the participants: *“There are not enough activity offers in the local community to all older adults. There are more groups now, exercise groups [...]. But there should also be other things...social things” (PT, 15).* Also, the HCPs stressed a need to provide continued individual PA support in the participant’s home: “*If they cannot get out from their home [ …*], *then they cannot attend to group exercises and such. Then they often remain sedentary in their home and keep deteriorating” (OT, 14).*

## Discussion

This study aimed to identify facilitators and barriers experienced by HCPs that influence the promotion of PA in older adults in the reablement context. The findings demonstrate that reablement is a heterogenic practice, influenced by several contextual factors and facilitators and barriers for promoting PA can be found at the participant, professional, organizational, and system level, as demonstrated in Fig. 1. The interrelationship between factors on all these levels influences HCPs’ abilities to promote PA by affecting their abilities to recruit appropriate participants, target the participants’ individual needs and goals, and support them in developing continued PA habits. The study findings add to the gap in knowledge regarding how PA can be appropriately integrated within real-life healthcare contexts [8]. They further identify several facilitators and barriers on different healthcare system levels, providing knowledge requested to inform the development of effective, meaningful, and integrated PA promotion strategies [8, 9].

The HCPs point out that the key facilitators and barriers for promoting PA are found within the individual participants and their environment. Similar to HCPs’ experiences in other reablement contexts [33], those in our study found that reablement participants constitute a heterogenic group with different values, motivations, and expectations. The HCPs find it important to consider these factors to promote PA in a meaningful and sustainable way to individual participants, which is in line with the WHO’s recommendation of individualizing PA promotion according to the individual’s healthcare needs, capacity, and preferences [7]. It has been emphasized that reablement should be person-centered [13, 15, 34–39]. Our findings demonstrate that individual participant factors are central to the HCPs’ approaches and that the participants’ individual goals represent an important and shared direction when developing reablement strategies with the participant. This is in line with principles of person-centered care, building upon therapeutic relationships between professionals, patients, and their significant others, which are built on mutual trust, understanding, and sharing collective knowledge [40]. Different individual factors on a participant level can explain why different strategies and approaches to PA promotion is used in reablement but do not explain the systematic differences between reablement settings, such as contextual differences in the emphasis on daily activities vs. exercises [19, 20] or individualized or standardized approaches [21], or differences in the degree to which promotion of PA is emphasized in reablement [13, 14, 19].

The study findings provide several potential explanations for the above mentioned differences. Firstly, at a participant level, our findings suggest that participants’ general characteristics may differ between reablement settings due to different recruitment strategies, the conceptualization of reablement, and needs in the particular municipality. As an example, the participants recruited may be more motivated to make an effort and engage in PA if they applied themselves, rather than if they were referred based on HCPs’ evaluation of their needs. Such differences in participant groups have previously been considered a challenge for developing a clear conceptualization of reablement [12, 13, 41] and may withhold important aspects to consider when discussing the appropriate conceptualization(s) of reablement. For example, one municipality in our study only included participants with a certain level of physical function, in which standardized exercise programs may be preferred by HCPs to meet similar needs between participants. Exercise programs were commonly included in reablement, though often requiring motivational support from HCPs. Emphasizing a meaningful introduction to why exercises are useful and external motivation to keep the participants’ motivation up has been recommended for promoting exercise [19], and reablement participants’ have indicated that they appreciate the physical strengthening and the ‘push’ they received in reablement [42] to be more physically active. However, the HCPs in our study emphasized that the incorporation of PA in daily life activities and building habits was essential to facilitate ongoing PA. PA incorporated in daily activities has been found equally effective as standardized exercise programs to improve function in reablement participants [43], and may enable a more person-centered approach to PA. This may enhance the participants’ perceived value of PA, by relating it to factors emphasized by older adults, such as social connections, meaningful activities, joy and fun [44].

Secondly, at a professional level, differences in the HCPs’ competencies, reablement philosophy, and interdisciplinary collaboration may lead to a different emphasis on PA promoting strategies. We found that some HCPs considered reablement to largely be equal to the promotion of PA, while other HCPs considered the promotion of PA to potentially be one of several approaches within reablement. Our findings suggest that the philosophies underpinning reablement differs between municipalities, drawing the reablement practice towards particular values, beliefs and priorities that may influence how PA is conceptualized and promoted in different settings. Ensuring sufficient competencies and motivation among home care staff has been considered essential in reablement [18, 33, 45, 46]. Our findings suggest that the reablement competencies of home care staff differ substantially between the municipalities, which requires HCPs to adapt their approaches to the home care staffs’ competency levels. The HCPs point out how simple, standardized PA programs may be required to ensure that home care staff can adequately follow up on the program, while more individually adapted approaches can be utilized by home care staff with reablement competencies and experience. However, while the emphasis on well-known exercises in some settings may enhance the home care staffs’ confidence, competencies and motivation to promote PA, it may also risk to devalue the reablement activities to instrumental, standardized tasks, that do not require the home care staffs’ professional competencies, and thus become uninspiring and demotivational. Unless such standardized exercises are introduced in a meaningful way, it may be contradictory to the goal-oriented and person-centered philosophy of reablement [13].

Thirdly, at an organizational level, we find that different ways of organizing reablement influence the degree to which the HCPs can adapt PA promotion strategies to the individual participant needs. In line with our findings, the available time for reablement delivery and interdisciplinary collaboration has been considered central to ensuring the quality of reablement [18, 21, 34, 36, 37, 47]. We found that there were substantial differences in the time available for reablement visits, which means that some HCPs need to rely on activities that can be efficiently performed in the participants’ home environment, while HCPs in other settings have the flexibility to also promote PA through outdoor and social activities. A lack of focus on outdoor and social activities in reablement has previously been demonstrated [48–50] and may be explained by such organizational differences. We do not believe that the findings of our study can inform any particular organizational model to be better suited to promote PA. Rather, we find that a number of organizational factors within each of the models have different influence on how PA is promoted and how it is targeted at individuals in a person-centered manner. The findings indicate that there is substantial variation within each of these organizational models and that attention need to be placed on how the interrelationship between these factors influences the HCPs judgements and practice.

Lastly, the HCPs also point out key mechanisms at a system level that influence how they can promote PA in a sustainable way. Having available and varied activity support in the community is considered important to support the participants to continue their activity habits after reablement, and the HCPs adapt their PA strategies accordingly. Also, having an overarching enablement philosophy in the municipal healthcare services is believed to be the key to reaching out to suitable people and delivering appropriate and continuous support for PA even beyond the period of reablement. Such changes in healthcare philosophy involving person-centered, integrated approaches that support people to maintain activity in older age are warranted through health policy [28, 51]. However, our findings suggest that the current organization of healthcare services creates central barriers for realizing this.

Our findings show that reablement is a multifaceted practice, highly dependent on the community context into which it is integrated. Previous research has shown a need to more clearly identify the characteristics of reablement and the appropriate target group of reablement, and further investigate critical components of reablement interventions [12, 13, 41]. However, based on our findings, we suggest that practical and research development of reablement should focus on it as an intervention at a participant level and consider it as an integrated care approach, involving multiple factors on a micro, meso, and macro level. Such a whole-system perspective is compatible with recent conceptualizations of evidence-based healthcare, showing the need to focus on the relationships between systems, individuals, and contextual factors across different settings to enable policy-makers and practitioners to make evidence-based decisions that are feasible, appropriate, meaningful, and effective [52].

### Strengths and weaknesses

A strength of this study is the purposeful sampling strategy used to ensure that we included HCPs from municipalities that differed from each other in the organization of reablement. This strategy enabled us to explore both similarities and differences in how the reablement context is experienced and how it influences HCPs’ practice across municipalities. Although the study findings relate to a Norwegian reablement setting, our study provides a potential frame of reference that can be used to explore contextual factors in other reablement settings, both nationally and internationally.

Also, we consider the interview guide and the semi-structured interview approach useful for capturing both the HCPs’ experiences with reablement in general and their experiences with PA promotion specifically. This approach enabled us to combine these experiences to gain a broad conceptual understanding of the facilitators and barriers in the reablement context, as seen through a micro, meso, and macro perspective of healthcare. A weakness of this study is that our recruitment strategy may have led to the inclusion of HCPs who are particularly enthusiastic about reablement, and we may not have addressed important facilitators and barriers experienced by HCPs who do not share this enthusiasm.

### Practical implications

These findings illustrate how different factors in an integrated healthcare system influence reablement delivery and can be a useful tool to further identify and evaluate factors that may influence reablement delivery in different contextual settings. This can inform clinicians, leaders, and politicians of the potentially successful factors and pitfalls that may enable or hinder successful implementation and delivery of reablement and/or strategies for promoting PA among older adults relative to the particular context.

### Research implications

The findings contribute to an increased understanding of factors influencing evidence-based healthcare in reablement from the HCPs’ perspective. The findings contribute to a greater understanding of mechanisms influencing reablement delivery in different contexts and demonstrate how the context withholds important mechanisms influencing how PA is promoted in reablement. There is a need to further explore how HCPs utilize and negotiate their professional competencies and perspectives within different reablement settings and how this influences how PA is promoted. Such different contextual mechanisms are important to acknowledge in future research of reablement and studies targeting PA promotion in older adults to develop evidence-based and person-centered real-life practice.

## Conclusion

The study findings demonstrate how several facilitators and barriers influence how HCPs can promote PA within the reablement context. We found that HCPs’ abilities to promote PA depended on an integrated coherence between factors at a participant, professional, organizational, and system level. These findings illustrate evidence from an HCP’s perspective and add to the understanding of how contextual factors influence reablement delivery, as well as facilitators and barriers for promoting PA in real-life healthcare settings. Our findings suggest that reablement may be a potentially suitable setting for promoting PA with older adults in an integrated and person-centered way, but that contextual factors on different levels need to be considered to meet needs and desires both on an individual and group level of older adults.

## Supplementary Information

**Additional file 1. ** Interview guide.

## Data Availability

The datasets generated and analyzed during the current study are not publicly available due to the risk of reducing the confidentiality of the study participants, but are available from the corresponding author on reasonable request.

## Abbreviations

PA: Physical activity
HCP: Health care professional
PT: Physiotherapist
OT: Occupational therapist
RN: Registered nurse
HT: Home trainer
ADL: Activities of daily living
