# Supplementary material for: Promotion of physical activity in older adults: facilitators and barriers experienced by healthcare personnel in the context of reablement
Source: BMC Health Serv Res. 2022 Jul 27;22:956. doi: 10.1186/s12913-022-08247-0 (PMC9327260; doi:10.1186/s12913-022-08247-0)
Supplement: Supplementary file 1 — Additional file 1. Interview guide. [file 12913_2022_8247_MOESM1_ESM.docx]

# Additional file 1: Interview guide

**PART 1 – general information about context and user group**

1. Can you describe—in overall terms—how you provide reablement in this municipality?
2. What is the typical content of the reablement intervention you provide?
3. What is your role in the reablement team?
4. Can you describe the group of people that receive reablement here?
5. What do you consider important to emphasize in reablement? Why?

**PART 2 – Physical activity**

1. Can you describe what you understand by the term physical activity?
2. Can you describe a situation where physical activity was part of the reablement? (*What did you do? What did the rest of the team do? Why did you do this?*

*Can you give some other examples? What did you do in those cases and why?)*

1. Can you describe a case, in which you think the physical activity was an important component of the reablement? *(Why was it important for this participant? What do you think, in general, influences the degree to which physical activity is important or not for the participants you meet in reablement? Why? If they answer that physical activity is not important, ask why this is.)*
2. Can you describe a case where physical activity contributed to the success of reablement? (*Why was it successful? Can you think of other cases where physical activity has been integrated in a successful way? Are there other factors than those you have mentioned by now that you find important for your ability to facilitate physical activity among the participants?*
3. Can you describe a situation in which it was difficult to facilitate physical activity?

*- Why was it difficult?*

*- Can you mention other situations in which it was difficult to facilitate physical activity?*

*- Are there other factors than those you have mentioned by now that can make it difficult to facilitate physical activity?*

1. The national health department recommends that older adults are physically active 150 minutes a week (+ repeat the rest of the recommendations). What do you think about these recommendations in the context of reablement?

CLOSING QUESTIONS

1. If you should point at anything that should be different for you to provide even better reablement than you do today, what could that be?
2. Repeat the aim of the interview. Ask if there is anything the interviewed person wants to add.
